# Supplementary material for: Retention of deposited ammonium and nitrate and its impact on the global forest carbon sink
Source: Nat Commun. 2022 Feb 15;13:880. doi: 10.1038/s41467-022-28345-1 (PMC8847626; doi:10.1038/s41467-022-28345-1)
Supplement: Supplementary file 1 — Supplementary Information [file 41467_2022_28345_MOESM1_ESM.pdf]

Supplementary Information

Retention of deposited ammonium and nitrate and its impact on the global forest carbon sinks

Geshere Abdisa Gurmesa[1, #], Ang Wang[1, 2, 3, #], Shanlong Li[1, 4], Shushi Peng[5 *], Wim de Vries[6], Per Gundersen[7], Philippe Ciais[8], Oliver L. Phillips[9], Erik A. Hobbie[10], Weixing Zhu[11], Knute Nadelhoffer[12], Yi Xi[5], Edith Bai[13], Tao Sun[1], Dexiang Chen[14], Wenjun Zhou[15], Yiping Zhang[15], Yingrong Guo[16], Jiaojun Zhu[1, 2], Lei Duan[17], Dejun Li[18], Keisuke Koba[19], Enzai Du[20], Guoyi Zhou[21], Xingguo Han[22], Shijie Han[23], and Yunting Fang[1, 2, 3 *]

[1]CAS Key Laboratory of Forest Ecology and Management, Institute of Applied Ecology, Chinese Academy of Sciences, Shenyang, China
[2]Qingyuan Forest CERN, Chinese Academy of Science, Shenyang, China
[3]Key Laboratory of Isotope Techniques and Applications, Shenyang, China
[4]Institute of Agricultural Resource and Environment, Jilin Academy of Agricultural Science, Changchun, China
[5]Sino-French Institute for Earth System Science, College of Urban and Environmental Sciences, Peking University, Beijing, China
[6]Wageningen University and Research, Environmental Systems Analysis Group, Wageningen, the Netherlands
[7]Department of Geosciences and Natural Resource Management, University of Copenhagen, Copenhagen, Denmark
[8]LSCE (CEA CNRS UVSQ UPSaclay) Centre d'Etudes Orme des Merisiers, Gifsur Yvette, France
[9]School of Geography, University of Leeds, UK
[10]Earth Systems Research Center, Morse Hall, University of New Hampshire, Durham, New Hampshire, USA
[11]Department of Biological Sciences, Binghamton University, The State University of New York, Binghamton, New York, USA
[12]Department of Ecology and Evolutionary Biology, University of Michigan, Ann Arbor, Michigan, USA
[13]School of Geographical Sciences, Northeast Normal University, Changchun, China
[14]Institute of Tropical Forestry, Chinese Academy of Forestry, Guangzhou, China
[15]CAS Key Laboratory of Tropical Forest Ecology, Xishuangbanna Tropical Botanical Garden, Chinese Academy of Sciences, Mengla, China
[16]Jiangxi Provincial Bureau of Forestry, Nanchang, Jiangxi, China
[17]State Key Laboratory of Environmental Simulation and Pollution Control, School of Environment, Tsinghua University, Beijing, China
[18]Institute of Subtropical Agriculture, Chinese Academy of Sciences, Changsha, China
[19]Center for Ecological Research, Kyoto University, Shiga, Japan
[20]State Key Laboratory of Earth Surface Processes and Resource Ecology, and Faculty of Geographical Science, Beijing Normal University, Beijing, China
[21]Institute of Ecology and School of Applied Meteorology, Nanjing University of Information Science and Technology, Nanjing, China
[22]State Key Laboratory of Vegetation and Environmental Change, Institute of Botany, Chinese Academy of Sciences, Beijing, China
[23]School of Life Sciences, Henan University, Kaifeng, China

[#]Equal contribution
*Corresponding author: Yunting Fang (fangyt@iae.ac.cn) and Shushi Peng (speng@pku.edu.cn)

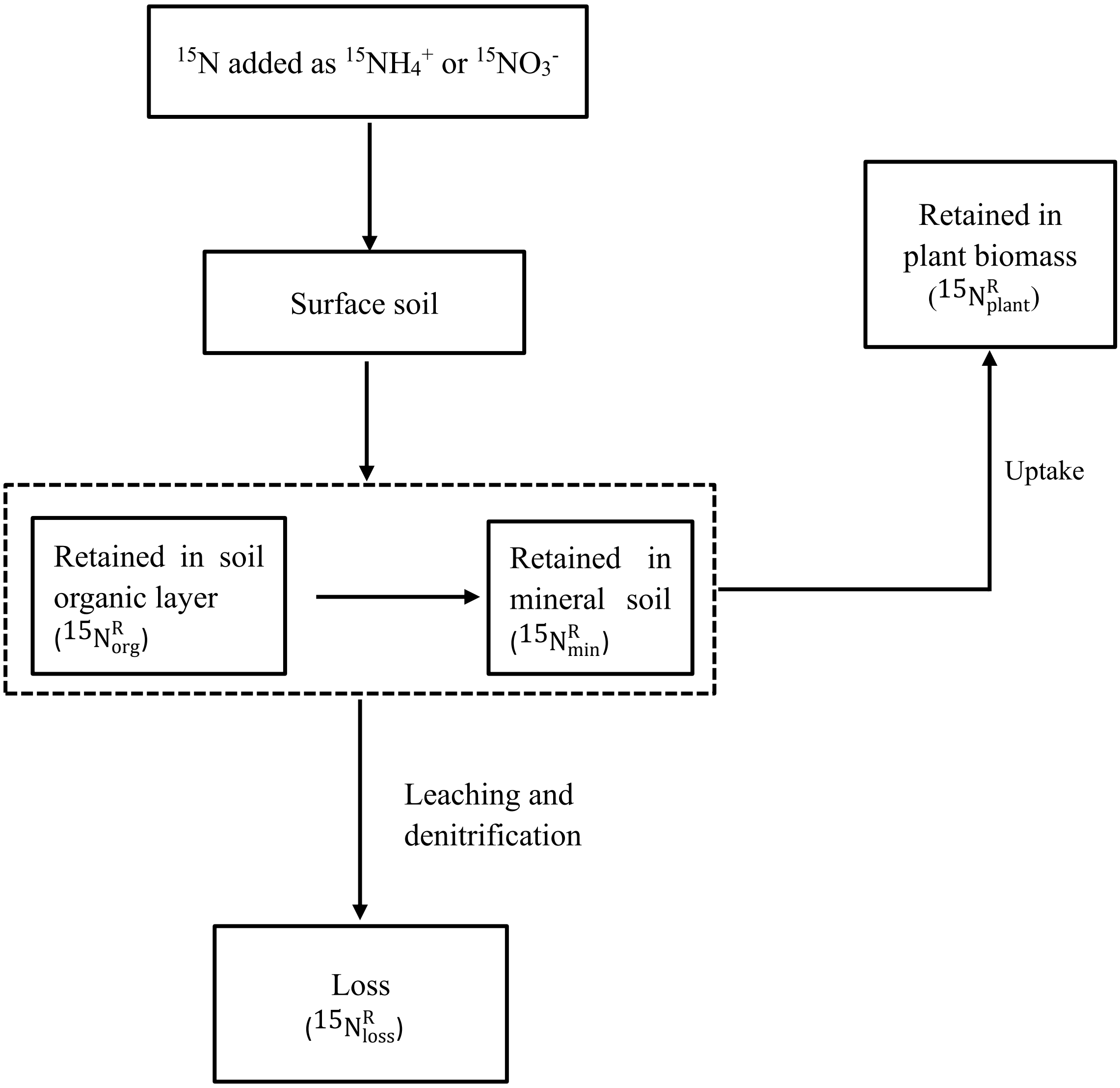

Supplementary Fig. 1. Fates and pathways of ^{15}N allocation into different pools after the ^{15}N-labelling indicating ^{15}N allocation fractions into plant ($^{15}N^R_{plant}$), soil organic layer ($^{15}N^R_{org}$), mineral soil ($^{15}N^R_{min}$), and leaching and gaseous losses ($^{15}N_{loss}$). We assumed total recovery of the added ^{15}N in combined measured pools (plant and soil) and fluxes (gaseous and leaching). Thus, $^{15}N^R_{plant} + {}^{15}N^R_{org} + {}^{15}N^R_{min} + {}^{15}N_{loss} = 100\%$. The fraction of ^{15}N that was not recovered in organic soil layer, mineral soil, and plant biomass (unrecovered fraction) is assumed to lost through leaching and gaseous pathways. The total loss fraction noted as $^{15}N_{loss}$ is predicted by mineral soil C/N ratio (Fig. 2a). Fraction of the added ^{15}N retained in soil organic matter ($^{15}N^R_{org}$) could be predicted by soil organic mass (Fig. 2b). Then, the remaining (100- ($^{15}N_{loss}$+$^{15}N^R_{org}$)) is assumed to be either retained the mineral soil or taken up by plants. The ratio of $^{15}N^R_{plant}$/ ($^{15}N^R_{plant}$+$^{15}N^R_{min}$) was predicted by net primary production (NPP) (Fig. 2c).

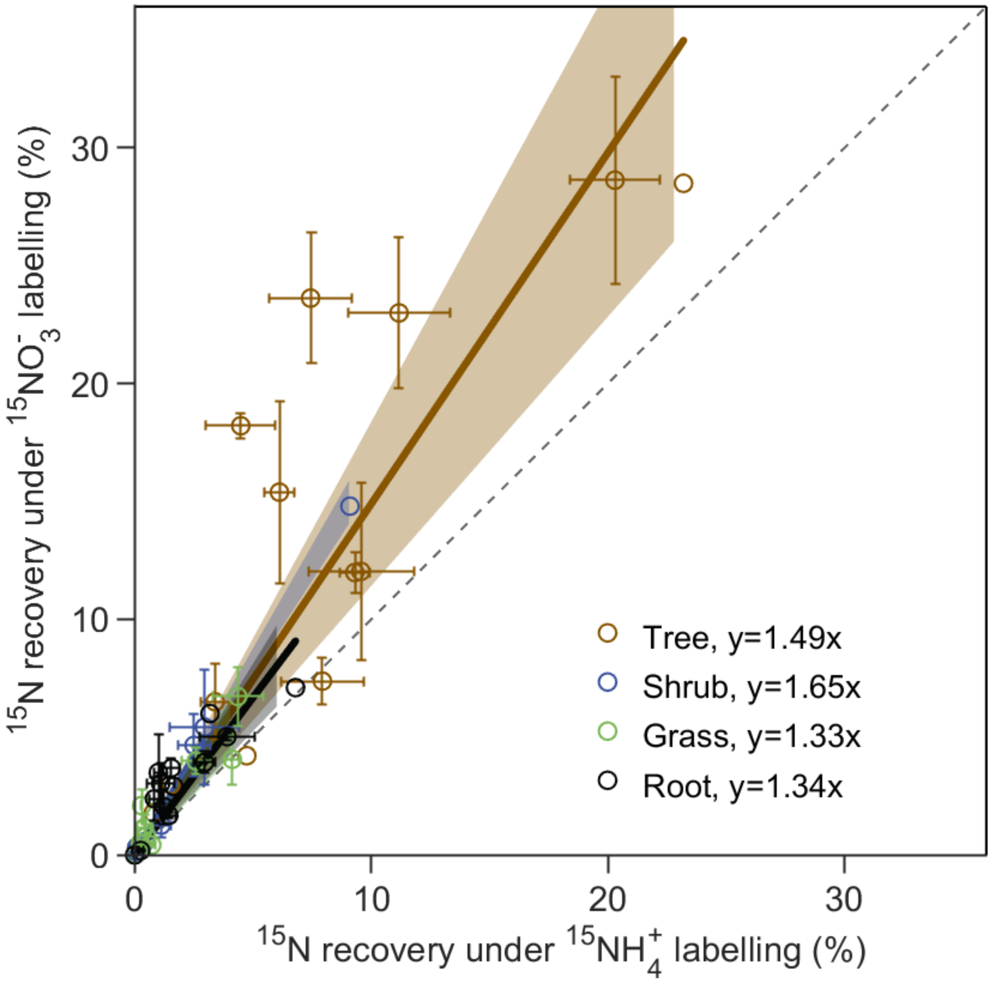

Supplementary Fig. 2. ^{15}N recoveries of $^{15}NH_4^+$ (x-axis) and $^{15}NO_3^-$ (y-axis) tracers in plant of different growth forms after ~1 year of paired ^{15}N-labelling. The black dashed line represents the 1:1 line. The shaded areas with corresponding colors indicate the 95% confidential intervals of linear regression for each pool. Data are presented as mean values +/- SE on both x-and y-axis.

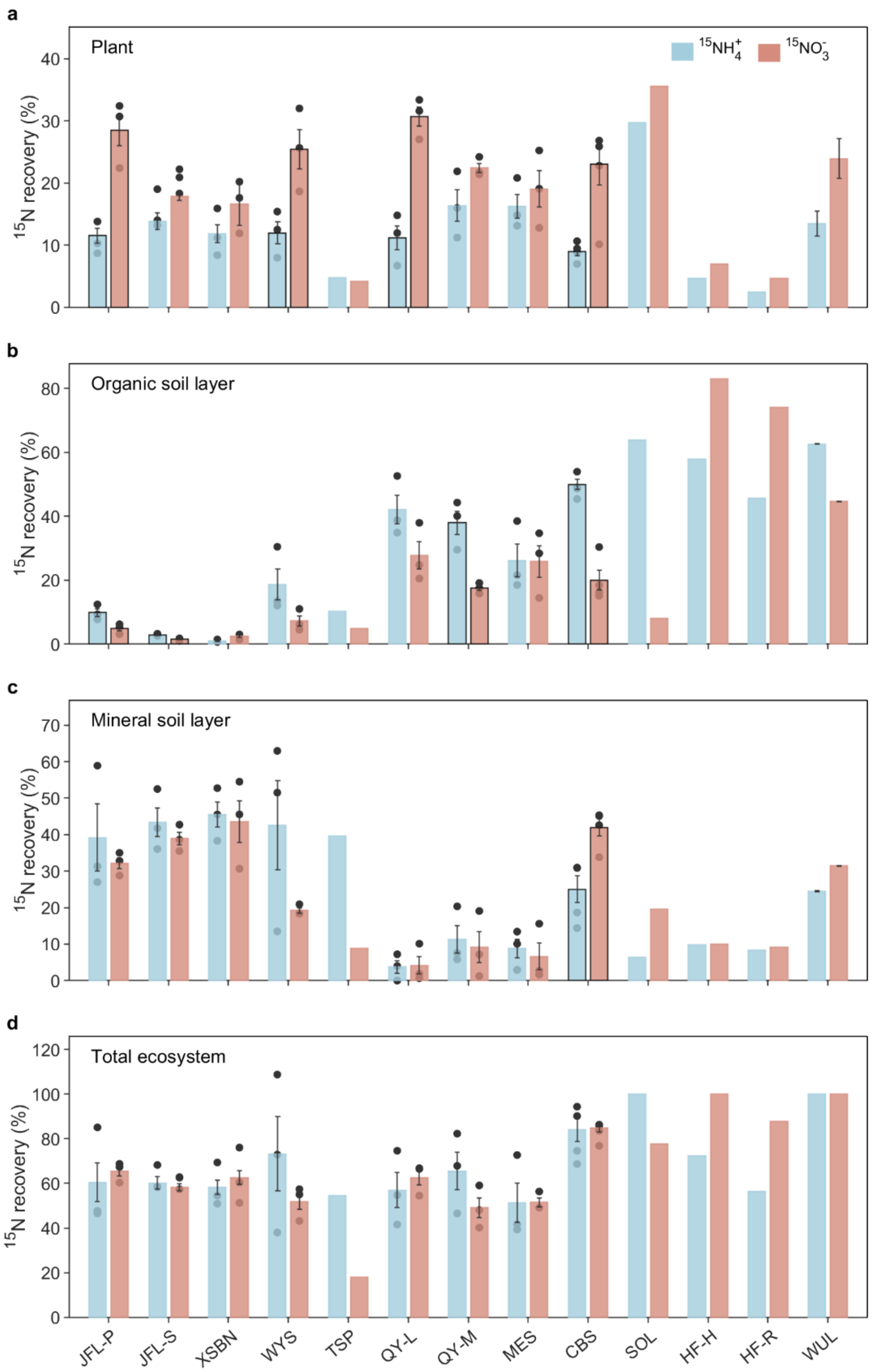

Supplementary Fig. 3. Percent recoveries of $^{15}NH_4^+$ and $^{15}NO_3^-$ tracer addition in **(a)** plants, **(b)** soil organic layer, **(c)** mineral soil, and **(d)** total ecosystem. JFL-P, Jianfengling-primary forest; JFL-S, Jianfengling-secondary forest; XSBN, Xishuangbanna; WYS, Wuyishan; TSP, Tieshanping; QY-L, Qingyuan-larch forest; QY-M, Qingyuan-mixed forest; MES, Maoershan; CBS, Changbai forest; SOL, Solling; HF-H, Harvard Forest-hardwood forest; HF-R, Harvard Forest-red pine forest; WUL,

Wülfersreuth. Data are presented as mean with error bars indicating SE for the Chinese sites (n = 4 CBS and n = 3 for the rest) and WUL (n = 5). Error bars are not shown for TSP, HF-H, HF-R, and SOL. Differences in ^{15}N recovery in ecosystem pools between $^{15}NH_4^+$-labelling and $^{15}NO_3^-$-labelling at each site were tested using paired t-test and black boarder line indicate sites with significant difference in recovery of $^{15}NO_3^-$ and $^{15}NH_4^+$ tracer in each compartment. For plant pools, recoveries were significantly higher for $^{15}NO_3^-$-labelling than for $^{15}NH_4^+$-labelling at JFL-P ($p = 0.007$), WYS ($p = 0.038$), QY-L ($p = 0.003$), and CBS ($p = 0.046$). In the organic soil layer, recoveries were significantly higher for $^{15}NH_4^+$-labelling than for $^{15}NO_3^-$-labelling at JFL-P ($p = 0.040$), JFL-S ($p = 0.014$), QY-M ($p = 0.010$), and CBS ($p < 0.001$). In the mineral soil, only the CBS had significantly higher recovery for $^{15}NO_3^-$-labelling than for $^{15}NH_4^+$-labelling ($p = 0.012$).

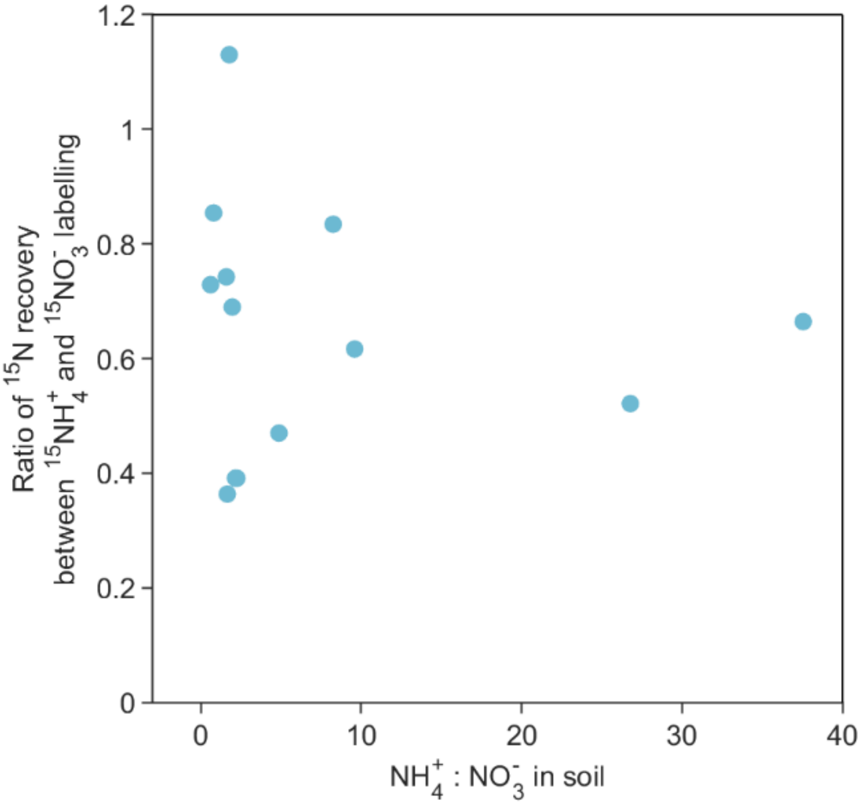

Supplementary Fig. 4. Relationship between relative recovery of $^{15}NH_4^+$ and $^{15}NO_3^-$ tracers (y-axis) in plant biomass and the soil ammonium to nitrate (NH_4^+:NO_3^-) ratio (x-axis). The NH_4^+:NO_3^- ratio is based on the concentration (mg N kg^{-1} soil) of extractable NH_4^+:NO_3^- in the top 10 cm mineral soil.

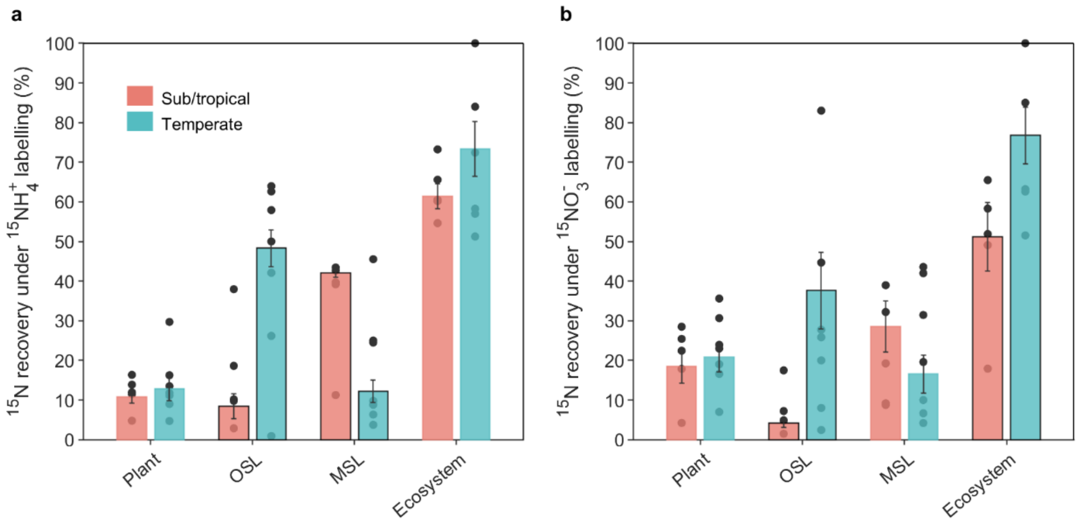

Supplementary Fig. 5. Percentage recoveries of the added ^{15}N tracer additions in plants, organic soil layer (OSL), mineral soil (MSL) and total ecosystem (Ecos.) in different forest biomes. **(a)** ^{15}N recoveries for $^{15}NH_4^+$-labelling, and **(b)** for $^{15}NO_3^-$-labelling. Data are presented as mean values with error bars indicating SE (n = 5 for tropical forest and n = 8 for temperate forests). Significant difference in ^{15}N recoveries between $^{15}NH_4^+$-labelling and $^{15}NO_3^-$-labelling in each ecosystem compartment is indicated black border lines. Recovery in OSL was significantly higher in temperate forests than in tropical forests for both $^{15}NH_4^+$ (paired t-test, p = 0.004) and $^{15}NO_3^-$-labelling (paired t-test, p = 0.009) while recovery in the MSL was significantly higher in tropical forests than in temperate forests for $^{15}NH_4^+$-labelling (paired t-test, p = 0.009).

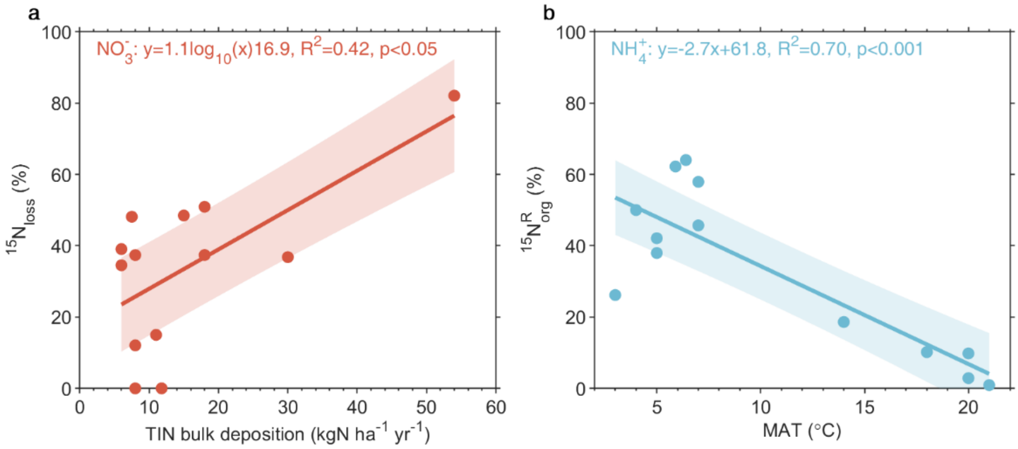

Supplementary Fig. 6. (a) The relationship between percentages of $^{15}NO_3^-$ loss ($^{15}N_{loss}$) and total inorganic N (TIN) bulk deposition, and **(b)** the relationship between percentages of $^{15}NH_4^+$ recovered in soil organic layer ($^{15}N^{R}_{org}$) and mean annual temperature (MAT). Data shown are mean values at each site. The corresponding shaded areas indicate the 95% confidence intervals of the linear regressions.

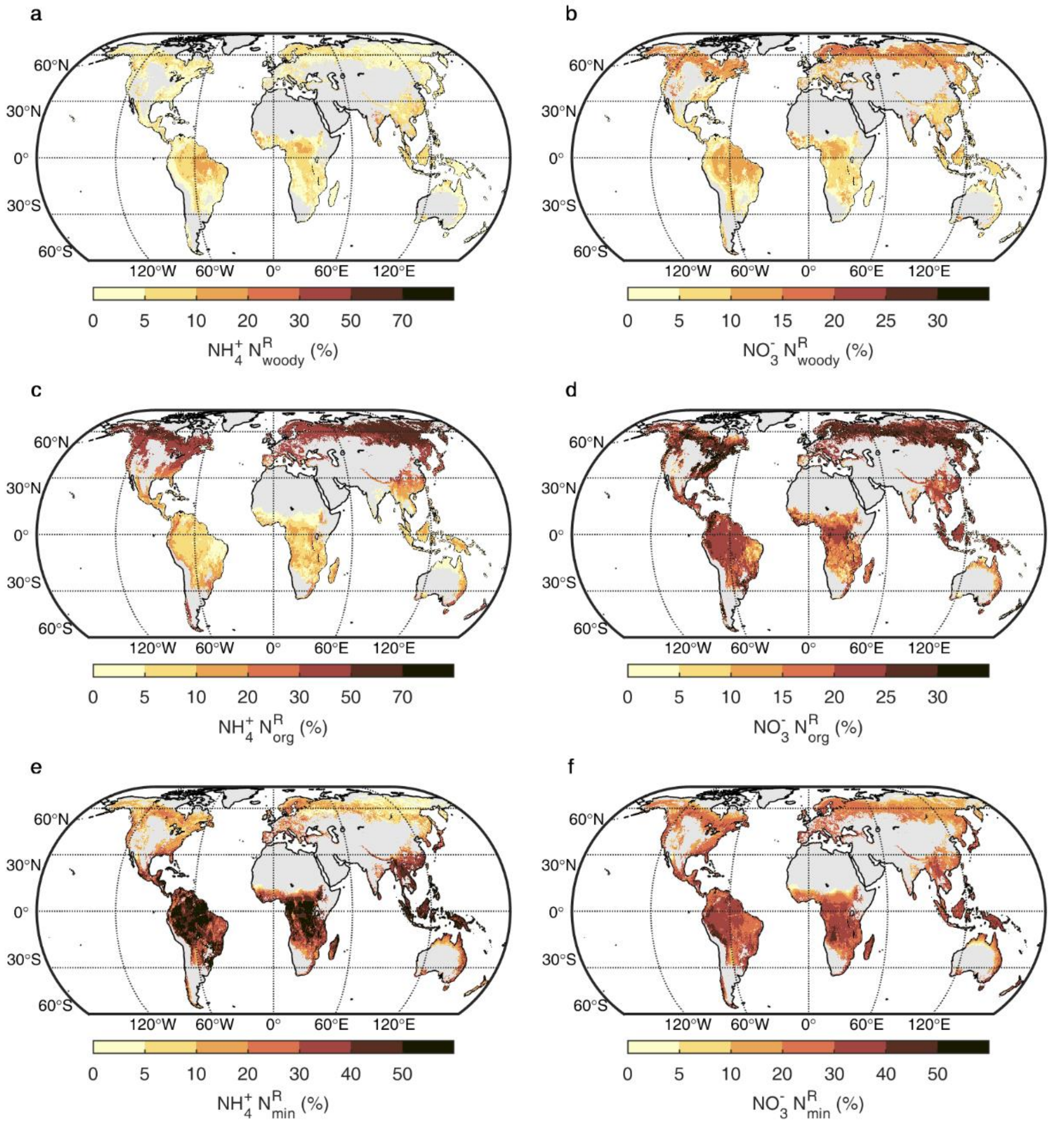

Supplementary Fig. 7. Estimated spatial patterns of ^{15}N retention (%) in plant and soil compartments. Figures a, c, and e show retention fraction in woody tree biomass ($^{15}\mathrm{N}^{\mathrm{R}}_{\mathrm{wood}}$), soil organic layer ($^{15}\mathrm{N}^{\mathrm{R}}_{\mathrm{org}}$), and mineral soil ($^{15}\mathrm{N}^{\mathrm{R}}_{\mathrm{min}}$), respectively for $^{15}NH_4^+$-labelling while b, f, and h show the retention in the corresponding ecosystem compartments for $^{15}NO_3^-$-labelling.

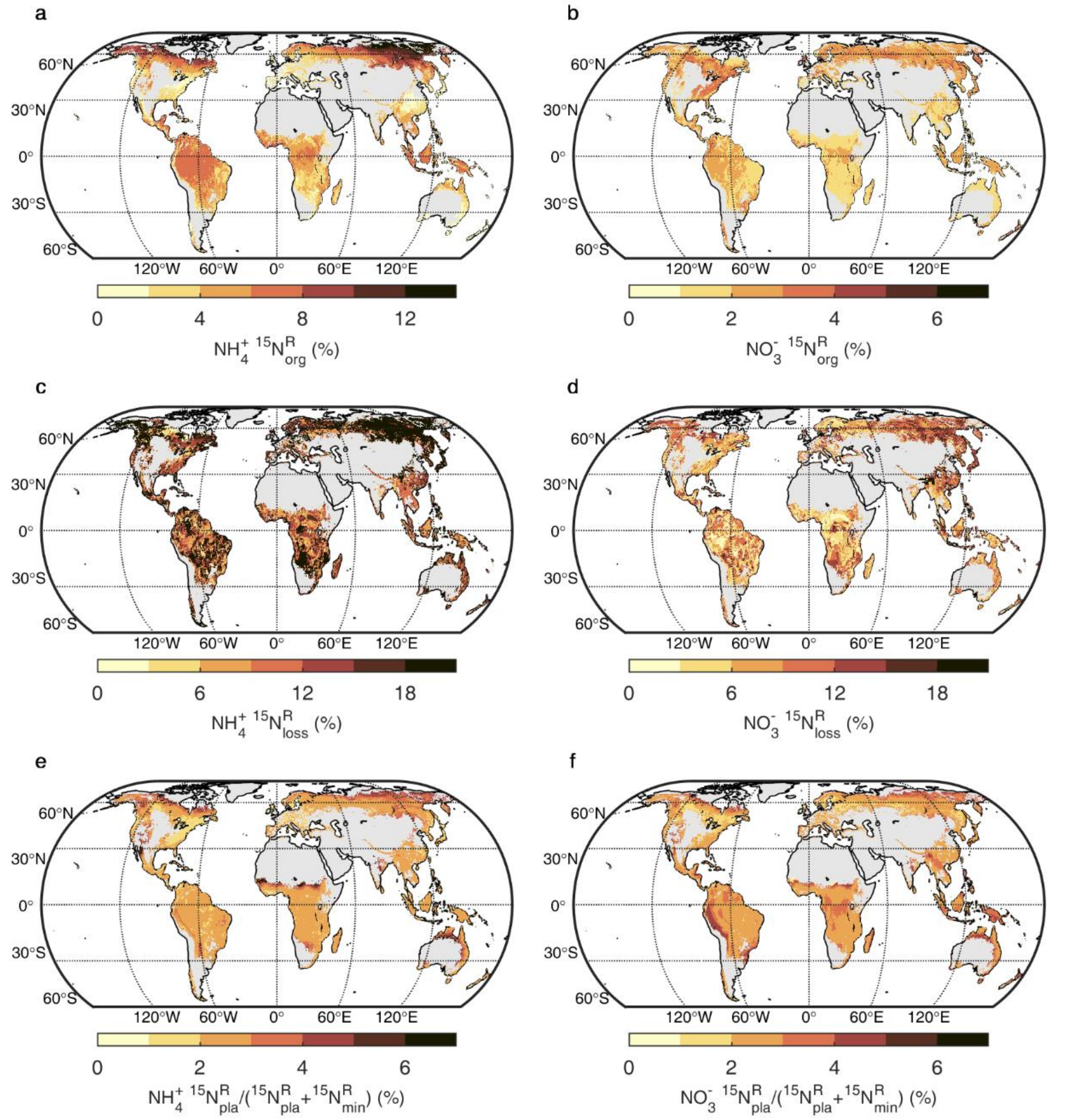

Supplementary Fig. 8. Spatial variation in the uncertainty of percent ^{15}N recovery in plant ($^{15}N^R_{pla}$), organic soil layer ($^{15}N^R_{org}$), mineral soil ($^{15}N^R_{min}$), and losses ($^{15}N^R_{loss}$). The uncertainties here include regression quantified using 1,000 random selections of 11 out of 13 sites and different input data for upscaling. Figures a, c, and e show uncertainty maps of $^{15}NH_4^+$-labelling while b, d, and f show uncertainty maps for $^{15}NO_3^-$-labelling.

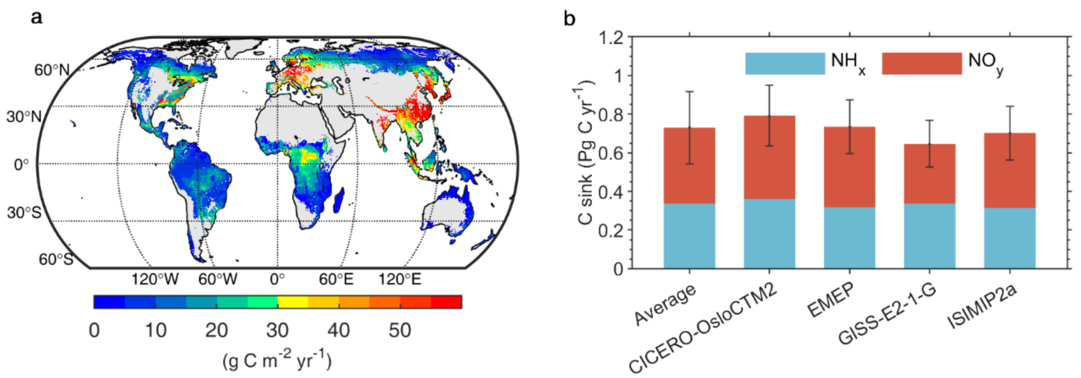

Supplementary Fig. 9. Estimates of C sink in global forests due to atmospheric N deposition. **(a)** Spatial patterns of forest C sink for the average N deposition (24.9 Tg N yr^{-1}); (**b)** Estimated mean global forest C sink induced by N deposition derived from four different models (Supplementary Table 5). Error bars in (b) indicate the 95% CIs (n = 60, derived from six C:N data × 1000 Monte Carlo 1000).

Supplementary Table 1. Site name, vegetation type, stand age, mean annual temperature (MAT), mean annual precipitation (MAP), and total N deposition at the 13 paired ^{15}N-labelling experiments used in this study. Total inorganic N depositions at the Chinese sites are based on 1-5 years of measurement of bulk or throughfall N input between 2005-2011. For the sites in Europe and US, N deposition is averaged from decades of throughfall measurements.

Site name	Location	Biome type	Tree species	Successional stage	Stand age (years)	MAT (ºC)	MAP (mm)	N deposition (kg N ha^{-1} yr^{-1})	Data source reference
Maoershan	127° 34'E, 45° 20'N	Temperate	Larch	Secondary forest	45	3	700	15	This study
Wuyishan	117° 46'E, 27° 51'N	Subtropical	Hemlock	Primary forest	150	14	2500	8	This study
Xishuangbanna	101° 15'E, 21° 56'N	Tropical	Mixed species	Primary forest	200	21	1593	8	This study
Jianfengling-secondary	108° 51'E, 18° 45'N	Tropical	Mixed species	Secondary forest	60	20	2449	6	Wang et al.[1]
Jianfengling-primary	108° 53'E, 18° 44'N	Tropical	Mixed species	Primary forest	200	20	2449	6	Wang et al.[2]
Tieshanping	106° 41'E, 29° 37'N	Subtropical	Mixed species	Primary forest	45	18	1105	54	Liu et al.[3]
Qingyuan - larch	124° 54'E, 41° 51'N	Temperate	Larch	Secondary forest	50	5	775	18	Li et al.[4]
Qingyuan - mixed	124° 55'E, 41° 51'N	Temperate	Mixed species	Secondary forest	50	5	775	18	Li et al.[4]
Changbai forest	128° 28'E, 42° 24'N	Temperate	Mixed species	Primary forest	150	4	745	11	Liu et al.[5]
Wülfersreuth	12° E, 50° N	Temperate	Norway spruce	Secondary forest	15	5.9	1072	12	Buchmann et al.[6]

Solling	9° 34' E, 51° 31'N	Temperate	Norway spruce	Secondary forest	75	6.4	1090	30	Feng et al.[7]
Harvard Forest -hardwood	72° 10'W, 42° 30'N	Temperate	Mixed species	Secondary forest	50	7	1120	8	Nadelhoffer et al.[8]
Harvard Forest -red pine	72° 10'W, 42° 30'N	Temperate	Red pine	Secondary forest	70	7	1120	8	Nadelhoffer et al.[8]

Supplementary Table 2. Selected soil characteristics at the 13 ^{15}N-labelling sites. Data sources are the same as in Supplementary Table 1.

Site name	Soil type	Bulk density (g cm^{-3})	Soil pH	Organic layer C/N	Mineral soil C/N	Total inorganic N (mg N kg^{-1} soil)	Soil NH_4^+/NO_3^-	Clay content (%)
Maoershan	Brown forest soil	0.90	5.3	24.8	10.2	32.5	0.8	26.6
Wuyishan	Mountainous yellow soil	0.50	3.7	27.3	16.1	5.9	4.9	24.8
Xishuangbanna	Latosols	1.14	5.0	35.8	9.0	7.4	2.0	30.2
Jianfengling-secondary forest	Lateritic yellow soil	1.08	4.1	36.4	11.3	4.4	1.6	32.7
Jianfengling-primary forest	Lateritic yellow soil	1.13	4.2	33.2	11.0	3.8	2.2	28.5
Tieshanping	Haplic acrisol	1.20	3.6	23.0	11.0	7.4	1.8	21.0
Qingyuan-larch	Brown forest soil	1.00	5.4	22.1	9.9	12.8	1.7	25.0
Qingyuan-mixed	Brown forest soil	0.70	5.8	21.9	8.9	15.8	0.6	25.4
Changbai forest	Brown forest soil	0.85	5.8	17.3	14.8	10.7	2.2	35.6
Wülfersreuth	Spodo-dystric cambisol		3.9	32.5	19.9	2.3	9.6	
Solling	Acidic dystric cambisol	0.97	3.0	25.0	22.0	7.4	8.3	18.5
Harvard Forest-hardwood	Inceptisols	0.67	3.8	27.0	20.6	3.5	37.6	9.0
Harvard Forest-red pine	Inceptisols	0.77	3.8	25.0	19.3	5.0	26. 8	

Supplementary Table 3. Experimental design (plot size, replication), forms of ^{15}N added, method of ^{15}N addition. At each site, the ^{15}N tracer was added above the forest floor, and the percent recovery of the added ^{15}N in major plant (tree compartments, understory vegetation and fine roots) and soil pools were determined approximately one year after the ^{15}N-labelling. The tracer was added once (pulse labelling) at all sites except at Solling and Harvard forests where the ^{15}N addition was repeated over time.

Site name	Plot size (m^2)	Replication	Forms of ^{15}N tracer added	^{15}N added (mg ^{15}N m^{-2})	Enrichment (% atom)	Labelling year	Sampling year
Maoershan	100	3	$^{15}NH_4NO_3$ or $NH_4{}^{15}NO_3$	25	99	May 2016	May 2017
Wuyishan	200	3	$^{15}NH_4NO_3$ or $NH_4{}^{15}NO_3$	25	99	April 2016	April 2017
Xishuangbanna	200	3	$^{15}NH_4NO_3$ or $NH_4{}^{15}NO_3$	25	99	April 2016	May 2017
Jianfengling-secondary	200	3	$^{15}NH_4NO_3$ or $NH_4{}^{15}NO_3$	25	99	April 2016	April 2017
Jianfengling-primary	200	3	$^{15}NH_4NO_3$ or $NH_4{}^{15}NO_3$	25	99	April 2015	April 2016
Tieshanping	144	3	$^{15}NH_4NO_3$ or $NH_4{}^{15}NO_3$	10-20	60	June 2012	Nov. 2013
Qingyuan-larch	200	3	$^{15}NH_4NO_3$ or $NH_4{}^{15}NO_3$	25	99	June 2014	June 2015
Qingyuan-mixed	200	3	$^{15}NH_4NO_3$ or $NH_4{}^{15}NO_3$	25	99	June 2014	June 2015
Changbai forest	200	3	$^{15}NH_4NO_3$ or $NH_4{}^{15}NO_3$	25	99	June 2014	July 2015
Wülfersreuth	40-70	5	$^{15}NH_4Cl$ or $K^{15}NO_3$	62	95-99	March 1991	November 1991
Solling	150	3	$^{15}NH_4NO_3$ or $NH_4{}^{15}NO_3$	46	95-98	2002/3/4*	Multiple times
Harvard Forest-hardwood	450	1	$^{15}NH_4Cl$ and $K^{15}NO_3$	28-35	99	1991-1992**	November 1992
Harvard Forest-red pine	450	1	$^{15}NH_4Cl$ and $K^{15}NO_3$	28-35	99	1991-1992**	November 1992

* The ^{15}N tracers were added multiple times in 2002, 2003, 2004 as a 1 mm rain event after every 30-40 mm of regular throughfall water collected.
**The ^{15}N tracers were applied to the ambient plots six times during the 1991 and 1992 growing seasons.

Supplementary Table 4. Potential predictors considered for regression analysis in the upscaling of ^{15}N recovery in plant and soil poos from the ^{15}N-labelling experiment to global N retention fraction and the summary statistics (R^2, p-value, BIC, AICc, VIF, Coefficient, and significance of important variables) for the best liner models selected for the upscaling. All possible models were fitted using glmulti (an R package for easy automated model selection with (generalized) linear models) and the model selection was conducted using based on corrected Akaike information criterion (AICc)[9]. Retention fractions in wood, total plant, organic soil layer, and mineral soil are indicated as $^{15}N^{R}_{wood}$, $^{15}N^{R}_{plant}$, $^{15}N^{R}_{org}$, $^{15}N^{R}_{min}$, respectively.

Variable	Potential predictors	Best model	R^2	*p*-value	BIC	AICc	VIF	Coefficients			Significance		
$^{15}NH_4^+$: $^{15}N^{R}_{org}$	MAP, MAT, NPP, Organic soil layer mass (OSL-M), Soil C/N, Leaf C/N	MAT + $\log_{10}$(OSL-M) (12 sites)	0.84	<0.001	96.0	99.8	1.9	Intercept<br>19.0	MAT<br>-1.5	$\log_{10}$(OSL-M)<br>22.4	Intercept<br>0.236	MAT<br>0.038	$\log_{10}$(OSL-M)<br>0.014
$^{15}NO_3^-$: $^{15}N^{R}_{org}$	MAP, MAT, NPP, Organic soil layer mass (M-SOM), Soil C/N, Leaf C/N	$\log_{10}$(OSL-M) (12 sites)	0.51	0.009	111.4	113.0		Intercept<br>-20.8	$\log_{10}$(OSL-M)<br>34.9		Intercept<br>0.216	$\log_{10}$(OSL-M)<br>0.009	
$^{15}NH_4^+$: $^{15}N^{R}_{loss}$	MAP, MAT, NPP, Organic soil layer mass (M-SOM), Soil C/N, Leaf C/N, Clay content, N deposition	Soil C/N + Clay content (12 sites)	0.51	0.040	97.9	101.7	2.8	Intercept<br>117.9	Soil C/N<br>-3.3	Clay content<br>-1.6	Intercept<br>0.003	Soil C/N<br>0.013	Clay content<br>0.037
$^{15}NO_3^-$: $^{15}N^{R}_{loss}$	MAP, MAT, NPP, Organic soil layer mass (OSL-M), Soil C/N, Leaf C/N, Clay content, N deposition	Soil C/N + Bulk N deposition (13 sites)	0.78	0.001	107.8	110.5	1.0	Intercept<br>45.4	Soil C/N<br>-1.9	Bulk N deposition<br>1.2	Intercept<br>0.001	Soil C/N<br>0.003	Bulk N deposition<br>0.001
$^{15}NH_4^+$: $^{15}N^{R}_{plant}$ / ($^{15}N^{R}_{plant}$+$^{15}N^{R}_{min}$)	MAP, MAT, NPP, Organic soil layer (OSL -M), Soil C/N, Leaf C/N	$\log_{10}$(NPP) + $\log_{10}$(OSL-M) (11 sites)	0.72	0.006	91.4	96.5	1.0	Intercept<br>206.9	$\log_{10}$(NPP)<br>-63.0	$\log_{10}$(OSL-M)<br>4.7	Intercept<br>0.001	$\log_{10}$(NPP)<br>0.003	$\log_{10}$(OSL-M)<br>0.517

$^{15}NO_3^-$: $^{15}N^R_{plant}$ / ($^{15}N^R_{plant}$+$^{15}N^R_{min}$)	MAP, MAT, NPP, Organic soil layer mass (OSL-M), Soil C/N, Leaf C/N	log_{10}(NPP) + log_{10}(OSL-M) (12 sites)	0.67	0.007	100.7	104.5	1.0	Intercept	log_{10}(NPP)	log_{10}(OSL-M)	Intercept	log_{10}(NPP)	log_{10}(OSL-M)
								221.1	-62.4	3.7	0.001	0.003	0.596
$^{15}NH_4^+$: $^{15}N^R_{wood}$ / ($^{15}N^R_{plant}$)	MAP, MAT, NPP, Organic soil layer mass (OSL-M), Soil C/N, Leaf C/N, Wood biomass	log_{10}(Wood biomass) (10 sites)	0.86	<0.001	73.7	76.8		Intercept	log_{10}(Wood biomass)		Intercept	log_{10}(Wood biomass)	
								-23.9	30.8		0.023	<0.001	
$^{15}NO_3^-$: $^{15}N^R_{wood}$ / ($^{15}N^R_{plant}$)	MAP, MAT, NPP, Organic soil layer mass (OSL-M), Soil C/N, Leaf C/N, Wood biomass	log_{10}(Wood biomass) (10 sites)	0.87	0.003	65.4	74.6	1.2	Intercept	log_{10}(Wood biomass)		Intercept	log_{10}(Wood biomass)	
								-23.8	29.2		0.000	<0.001	

Supplementary Table 5. Model estimates of N deposition (Tg N yr^{-1}) on global boreal, temperate, and tropical forests in reduced (NH_x) and oxidized (NO_y) forms. Estimates are shown for the year 2000 for CICERO-OsloCTM2[10], and GISS-E2-1-G[11] and for the year 2010 for EMEP[12] and THQ[13] models.

Biome type	Area (million km^2)	Models			
		CICERO-OsloCTM2	GISS-E2-1-G	THQ	EMEP
**NH_x**					
Boreal forests	12	2.6	1.6	2.5	1.1
Temperate forests	7	3.4	5.6	3.1	5.2
Tropical forests	23	8.2	7.1	6.8	7.6
**Subtotal**		14.2	14.3	12.5	14.0
**NO_y**					
Boreal forests	12	2.3	1.2	2.0	1.5
Temperate forests	7	3.7	3.4	3.7	4.2
Tropical forests	23	5.4	4.9	4.8	7.7
**Subtotal**		11.5	9.4	10.5	13.3
**Total (NH_x + NO_y)**	**42**	**25.7**	**23.7**	**23.0**	**27.3**

Supplementary Table 6. Mean deposited NH_x and NO_y (Tg N yr^{-1}) and their retentions in plant, organic layer, and mineral soil pools of global boreal, temperate, and tropical forests. For each biome, N retention fraction (%) is upscaled based on the 13 ^{15}N-labelling experiments as described in the method. Then, N retention (Tg N yr^{-1}) is calculated based on the average NH_x and NO_y deposition (Supplementary Table 5) and the retention fractions. Superscripts and subscripts after mean values indicate 95% confidence intervals.

Biome type	N deposition (Tg N yr^{-1})	N retention (Tg N yr^{-1})			
		Total plant biomass	Woody biomass	Organic layer	Mineral soil
**NH_x**					
Boreal forests	2.0	$0.21^{0.47}_{0.05}$	$0.08^{0.18}_{0.02}$	$0.91^{1.44}_{0.45}$	$0.26^{0.53}_{0.08}$
Temperate forests	4.3	$0.54^{0.89}_{0.24}$	$0.22^{0.37}_{0.10}$	$1.17^{1.48}_{0.87}$	$1.16^{1.91}_{0.54}$
Tropical forests	7.4	$1.26^{1.72}_{0.83}$	$0.42^{0.57}_{0.27}$	$0.75^{1.74}_{0.20}$	$3.17^{4.15}_{2.20}$
**Subtotal**	13.8	$2.00^{2.69}_{1.34}$	$0.72^{0.96}_{0.47}$	$2.83^{3.55}_{2.26}$	$4.59^{5.95}_{3.28}$
**NO_y**					
Boreal forests	1.7	$0.61^{0.93}_{0.36}$	$0.22^{0.34}_{0.13}$	$0.45^{0.65}_{0.23}$	$0.41^{0.61}_{0.23}$
Temperate forests	3.7	$0.87^{1.11}_{0.63}$	$0.30^{0.39}_{0.22}$	$0.87^{1.09}_{0.66}$	$0.97^{1.23}_{0.71}$
Tropical forests	5.7	$1.38^{2.07}_{0.97}$	$0.43^{0.64}_{0.29}$	$1.11^{1.64}_{0.81}$	$1.82^{2.67}_{1.32}$
**Subtotal**	11.2	$2.86^{3.73}_{2.05}$	$0.95^{1.24}_{0.68}$	$2.42^{3.09}_{1.78}$	$3.20^{4.27}_{2.36}$
**Total (NH_x + NO_y)**	24.9	$4.86^{6.22}_{3.62}$	$1.67^{2.12}_{1.23}$	$5.25^{6.19}_{4.48}$	$7.79^{9.82}_{5.90}$

Supplementary Table 7. Contribution of deposited NH_x and NO_y to C sink (Pg C yr^{-1}) in trees (woody part), organic layer, and mineral soil of global boreal, temperate, and tropical forests. For C sink in soil the assumes that 80% of the N retained in both organic layer and mineral soil are immobilized in soil organic matter. Carbon gain per unit N deposited was calculated by diving the estimated C sink by N deposition. Superscripts and subscripts after mean values indicate 95% confidence intervals.

Biome type	C sink (Pg C yr^{-1})				Carbon gain per unit N deposited (kg C kg^{-1} N)			
	Tree	Organic layer	Mineral soil	Total	Tree	Organic layer	Mineral soil	Total
**NHx**								
Boreal forests	$0.03_{0.01}^{0.07}$	$0.04_{0.02}^{0.06}$	$0.00_{0.00}^{0.01}$	$0.07_{0.03}^{0.13}$	14_{3}^{37}	18_{9}^{29}	2_{1}^{5}	35_{22}^{54}
Temperate forests	$0.05_{0.02}^{0.09}$	$0.05_{0.04}^{0.06}$	$0.01_{0.01}^{0.02}$	$0.11_{0.07}^{0.16}$	11_{4}^{20}	11_{8}^{14}	3_{1}^{5}	26_{18}^{38}
Tropical forests	$0.09_{0.04}^{0.16}$	$0.03_{0.01}^{0.06}$	$0.03_{0.02}^{0.04}$	$0.15_{0.10}^{0.22}$	12_{6}^{22}	4_{1}^{9}	4_{3}^{6}	21_{14}^{29}
**Subtotal**	$0.17_{0.08}^{0.27}$	$0.11_{0.09}^{0.14}$	$0.05_{0.04}^{0.07}$	$0.33_{0.23}^{0.45}$	12_{5}^{20}	8_{7}^{10}	4_{3}^{5}	24_{17}^{33}
**NOy**								
Boreal forests	$0.08_{0.03}^{0.15}$	$0.02_{0.01}^{0.03}$	$0.01_{0.00}^{0.01}$	$0.10_{0.05}^{0.18}$	45_{17}^{84}	10_{6}^{15}	4_{2}^{7}	59_{35}^{86}
Temperate forests	$0.08_{0.04}^{0.14}$	$0.04_{0.03}^{0.05}$	$0.01_{0.01}^{0.01}$	$0.13_{0.08}^{0.19}$	21_{10}^{37}	10_{8}^{13}	3_{2}^{4}	34_{22}^{50}
Tropical forests	$0.10_{0.05}^{0.18}$	$0.04_{0.03}^{0.06}$	$0.02_{0.01}^{0.03}$	$0.16_{0.10}^{0.26}$	17_{8}^{32}	7_{5}^{11}	3_{2}^{5}	28_{19}^{39}
**Subtotal**	$0.25_{0.12}^{0.38}$	$0.10_{0.07}^{0.12}$	$0.04_{0.03}^{0.05}$	$0.39_{0.24}^{0.53}$	23_{11}^{34}	9_{6}^{11}	3_{2}^{4}	35_{23}^{46}
**Total (NH_x + NO_y)**	$0.42_{0.20}^{0.64}$	$0.21_{0.18}^{0.25}$	$0.09_{0.07}^{0.11}$	$0.72_{0.49}^{0.96}$	17_{8}^{26}	8_{7}^{10}	4_{3}^{4}	29_{20}^{38}

Supplementary Table 8. Average values of wood C/N ratio for needle forest (NF), evergreen broad-leaved forest (EBF), deciduous broad-leaved forest (DBF), and mixed forest (MXF) used to estimate C sink in global forests due to N deposition.

Dataset source	NF	EBF	DBF	MXF	Reference
GOLUM	525	148	471	336.5	Wang et al[14]
Cleveland et al	250	150	175	175	Cleveland et al[15]
Du and de Vries	500	250	280	375	Du and de Vrie[16]
CABLE	482	270	312	376	Haverd et al 2018[17]
ORCHIDEE-min	110	110	110	110	Sun et al[18] and Zaehle et al[19]
ORCHIDEE-max	517	414	414	414	Sun et al[18] and Zaehle et al[19]

Supplementary Table 9. Global average total N deposition (Tg N yr^{-1}) on forests, its contribution to C sink (Pg C yr^{-1}), and C gain per unit N deposited (kg C kg^{-1} N) in global forests as estimated by various studies using stoichiometric scaling, inventory data (extrapolation), and model analyses. The global average C gain per unit N deposited (kg C kg^{-1} N) was calculated by dividing the global C sink by the global N deposition. [1]the numbers indicate the order in Fig. 4.

Nr[1]	Study	Method used to estimate C sink	Ecosystem	Total area (million km^2)	N deposition (Tg N yr^{-1})	Reference period/year	C sink (Pg C yr^{-1})	C gain per unit N deposited (kg C kg^{-1} N)
	This study	Stoichiometric scaling	Forest	42	24.9	2010	0.72	29
R1	De Vries et al.[20]	Stoichiometric scaling	Forest	32.6	15.3	1993	0.35	23
R2	Du and de Vries[16]	Stoichiometric scaling	Forest	40.6	21	2001	0.25	12
R3	Wang et al.[21]	Stoichiometric scaling	Forest	26	11	1997-2013	0.27	25
R4	Nadelhoffer et al.[22]	Stoichiometric scaling	Forest	-	5.1	1990s	0.25	49
R5	Thomas et al.[23]	Extrapolation based on inventory data	Forest	-	5.1	1980s-1990s	0.31	61
R6	Schulte-Uebbing and De Vries [24]	Meta-analysis	Forest		15		0.15	10
R7	Fleischer et al.[25]	Model analyses	Forest	38.5	20	1900-2007	0.46	23
	Churkina et al.[26]	Model analyses	Terrestrial			1990s	1.02	
	Churkina et al.[27]	Model analyses	Terrestrial			1981-2000	0.69	
	O'Sullivan et al.[28]	Model analyses	Terrestrial			2010-2016	0.69	
	Devaraju et al.[29]	Model analyses	Terrestrial			1970s-2000s	0.58	
	Jain et al.[30]	Model analyses	Terrestrial			1990s	0.26	
	Thornton et al.[31]	Model analyses	Terrestrial			1981-2000	0.24	
	Zaehle[32]	Model analyses	Terrestrial			1990	0.20	
	Bonan and Levis[33]	Model analyses	Terrestrial			1973-2004	0.19	
	Bala et al.[34]	Model analyses	Terrestrial			1906-2006	0.17	

References

1. Wang, A. *et al.* Dynamics and multi-annual fate of atmospherically deposited nitrogen in montane tropical forests. *Glob. Chang. Biol.* **27**, 2076-2087 (2021).
2. Wang, A. *et al.* Fates of atmospheric deposited nitrogen in an Asian tropical primary forest. *For. Ecol. Manage.* **411**, 213-222 (2018).
3. Liu, W. *et al.* In situ ^{15}N labeling experiment reveals different long-term responses to ammonium and nitrate inputs in N-saturated subtropical forest. *J. Geophys. Res. Biogeosciences* **122**, 2251-2264 (2017).
4. Li, S. *et al.* Fate of atmospherically deposited NH_4^+ and NO_3^- in two temperate forests in China: temporal pattern and redistribution. *Ecol. Appl.* **0**, e01920 (2019).
5. Liu, J. *et al.* Different fates of deposited NH_4^+ and NO_3^- in a temperate forest in northeast China: a ^{15}N tracer study. *Glob. Chang. Biol.* **23**, 2441-2449 (2017).
6. Buchmann, N., Gebauer, G. & Schulze, E. D. Partitioning of ^{15}N-labeled ammonium and nitrate among soil, litter, below- and above-ground biomass of trees and understory in a 15-year-old Picea abies plantation. *Biogeochemistry* **33**, 1-23 (1996).
7. Feng, Z., Brumme, R., Xu, Y. J. & Lamersdorf, N. Tracing the fate of mineral N compounds under high ambient N deposition in a Norway spruce forest at Solling/Germany. *For. Ecol. Manage.* **255**, 2061-2073 (2008).
8. Nadelhoffer, K. J., Downs, M. R. & Fry, B. Sinks for ^{15}N-enriched additions to an oak forest and a red pine plantation. *Ecol. Appl.* **9**, 72-86 (1999).
9. Calcagno, V. & de Mazancourt, C. glmulti: an R package for easy automated model selection with (generalized) linear models. J. Stat. Softw. 34, 1-29 (2010)
10. Skeie, R. B. *et al.* Black carbon in the atmosphere and snow, from pre-industrial times until present. *Atmos. Chem. Phys.* (2011) doi:10.5194/acp-11-6809-2011.
11. NASA Goddard Institute for Space Studies (NASA/GISS). NASA-GISS GISS-E2.1G model output prepared for CMIP6 CMIP. Version 20180827. (2018) doi:10.22033/ESGF/CMIP6.1400.
12. Schwede, D. B. *et al.* Spatial variation of modelled total, dry and wet nitrogen deposition to forests at global scale. *Environ. Pollut.* **243**, 1287-1301 (2018).
13. Tian, H. *et al.* The global N_2O model intercomparison project. *Bull. Am. Meteorol. Soc.* **99**, 1231-1251 (2018).
14. Wang, Y. *et al.* GOLUM-CNP v1.0: A data-driven modeling of carbon, nitrogen and phosphorus cycles in major terrestrial biomes. *Geosci. Model Dev.* **11**, 3903-3928 (2018).
15. Cleveland, C. C. *et al.* Patterns of new versus recycled primary production in the terrestrial biosphere. *Proc. Natl. Acad. Sci. U. S. A.* **110**, 12733-12737 (2013).
16. Du, E. & de Vries, W. Nitrogen-induced new net primary production and carbon sequestration in global forests. *Environ. Pollut.* **242**, 1476-1487 (2018).
17. Haverd, V. *et al.* A new version of the CABLE land surface model (Subversion revision r4601) incorporating land use and land cover change, woody vegetation demography, and a novel optimisation-based approach to plant coordination of

photosynthesis. *Geosci. Model Dev.* **11**, 2995-3026 (2018).

18. Sun, Y. *et al.* Global evaluation of nutrient enabled version land surface model ORCHIDEE-CNP v1.2 (r5986). *Geosci. Model Dev. Discuss.* **14**, 1987-2010 (2020).
19. Zaehle, S. & Friend, A. D. Carbon and nitrogen cycle dynamics in the O-CN land surface model: 1. Model description, site-scale evaluation, and sensitivity to parameter estimates. *Global Biogeochem. Cycles* **24**, GB1005 (2010).
20. de Vries, W., Du, E. & Butterbach-Bahl, K. Short and long-term impacts of nitrogen deposition on carbon sequestration by forest ecosystems. Curr. Opin. Environ. Sustain. **9-10**, 90-104 (2014).
21. Wang, R. *et al.* Global forest carbon uptake due to nitrogen and phosphorus deposition from 1850 to 2100. *Glob. Chang. Biol.* **23**, 4854-4872 (2017).
22. Nadelhoffer, K. J. *et al.* Nitrogen deposition makes a minor contribution to carbon sequestration in temperate forests. *Nature* **398**, 145-148 (1999).
23. Thomas, R. Q., Canham, C. D., Weathers, K. C. & Goodale, C. L. Increased tree carbon storage in response to nitrogen deposition in the US. *Nat. Geosci.* **3**, 13-17 (2010).
24. Schulte-Uebbing, L. & de Vries, W. Global-scale impacts of nitrogen deposition on tree carbon sequestration in tropical, temperate, and boreal forests: A meta-analysis. *Glob. Chang. Biol.* **24**, E416-E431 (2018).
25. Fleischer, K. *et al.* Low historical nitrogen deposition effect on carbon sequestration in the boreal zone. *J. Geophys. Res. Biogeosciences* **120**, 2542-2561 (2015).
26. Churkina, G. *et al.* Synergy of rising nitrogen depositions and atmospheric CO_2 on land carbon uptake moderately offsets global warming. *Global Biogeochem. Cycles* **23**, GB4027 (2009).
27. Churkina, G., Trusilova, K., Vetter, M. & Dentener, F. Contributions of nitrogen deposition and forest regrowth to terrestrial carbon uptake. *Carbon Balance Manag.* **2**, 5 (2007).
28. O'Sullivan, M. *et al.* Have synergies between nitrogen deposition and atmospheric CO_2 driven the recent enhancement of the terrestrial carbon sink? *Global Biogeochem. Cycles* **33**, 163-180 (2019).
29. Devaraju, N., Bala, G., Caldeira, K. & Nemani, R. A model based investigation of the relative importance of CO_2-fertilization, climate warming, nitrogen deposition and land use change on the global terrestrial carbon uptake in the historical period. *Clim. Dyn.* **47**, 173-190 (2016).
30. Jain, A. *et al.* Nitrogen attenuation of terrestrial carbon cycle response to global environmental factors. *Global Biogeochem. Cycles* **23**, GB4028 (2009).
31. Thornton, P. E., Lamarque, J. F., Rosenbloom, N. A. & Mahowald, N. M. Influence of carbon-nitrogen cycle coupling on land model response to CO_2 fertilization and climate variability. *Global Biogeochem. Cycles* **21**, GB4018 (2007).
32. Zaehle, S. Terrestrial nitrogen-carbon cycle interactions at the global scale. *Philos. Trans. R. Soc. B Biol. Sci.* **368**, 20130125 (2013).

33. Bonan, G. B. & Levis, S. Quantifying carbon-nitrogen feedbacks in the Community Land Model (CLM4). *Geophys. Res. Lett.* **37**, L07401 (2010).
34. Bala, G., Devaraju, N., Chaturvedi, R. K., Caldeira, K. & Nemani, R. Nitrogen deposition: How important is it for global terrestrial carbon uptake. *Biogeosciences* **10**, 7147-7160 (2013).
