# Supplementary material for: Retention of deposited ammonium and nitrate and its impact on the global forest carbon sink
Source: Nat Commun. 2022 Feb 15;13:880. doi: 10.1038/s41467-022-28345-1 (PMC8847626; doi:10.1038/s41467-022-28345-1)
Supplement: Supplementary file 3 — Reporting Summary [file 41467_2022_28345_MOESM3_ESM.pdf]

nature portfolio

Corresponding author(s): Yunting Fang
Shushi Peng

Last updated by author(s): Nov 23, 2021

Reporting Summary

Nature Portfolio wishes to improve the reproducibility of the work that we publish. This form provides structure for consistency and transparency in reporting. For further information on Nature Portfolio policies, see our Editorial Policies and the Editorial Policy Checklist.

Statistics

For all statistical analyses, confirm that the following items are present in the figure legend, table legend, main text, or Methods section.

n/a	Confirmed	
☐	☒	The exact sample size (*n*) for each experimental group/condition, given as a discrete number and unit of measurement
☒	☐	A statement on whether measurements were taken from distinct samples or whether the same sample was measured repeatedly
☐	☒	The statistical test(s) used AND whether they are one- or two-sided<br>*Only common tests should be described solely by name; describe more complex techniques in the Methods section.*
☒	☐	A description of all covariates tested
☐	☒	A description of any assumptions or corrections, such as tests of normality and adjustment for multiple comparisons
☐	☒	A full description of the statistical parameters including central tendency (e.g. means) or other basic estimates (e.g. regression coefficient) AND variation (e.g. standard deviation) or associated estimates of uncertainty (e.g. confidence intervals)
☐	☒	For null hypothesis testing, the test statistic (e.g. *F*, *t*, *r*) with confidence intervals, effect sizes, degrees of freedom and *P* value noted<br>*Give P values as exact values whenever suitable.*
☒	☐	For Bayesian analysis, information on the choice of priors and Markov chain Monte Carlo settings
☒	☐	For hierarchical and complex designs, identification of the appropriate level for tests and full reporting of outcomes
☒	☐	Estimates of effect sizes (e.g. Cohen's *d*, Pearson's *r*), indicating how they were calculated

Our web collection on statistics for biologists contains articles on many of the points above.

Software and code

Policy information about availability of computer code

Data collection	No software was used to collect data.
Data analysis	The statistic analyses and mapping were both performed using MATLAB (R2020a) and R Studio (version 1.3.959).

For manuscripts utilizing custom algorithms or software that are central to the research but not yet described in published literature, software must be made available to editors and reviewers. We strongly encourage code deposition in a community repository (e.g. GitHub). See the Nature Portfolio guidelines for submitting code & software for further information.

Data

Policy information about availability of data

All manuscripts must include a data availability statement. This statement should provide the following information, where applicable:

- Accession codes, unique identifiers, or web links for publicly available datasets
- A description of any restrictions on data availability
- For clinical datasets or third party data, please ensure that the statement adheres to our policy

The N retention and C sink data generated during and/or analysed during the current study are available in the Dryad Digital Repository (DOI: 10.5061/dryad.cfxpnvx3d).
The MODIS NPP product (MOD17, version 5.5) is downloaded from https://lpdaac.usgs.gov/products/mod17a2hv061/.
The C/N ratios of mineral soil at 0-10 cm were obtained from Harmonized World Soil Database (https://www.isric.org/documents/document-type/isric-report-201201-isric-wise-derived-soil-properties-5-5-arc-minutes), BNU soil dataset (http://globalchange.bnu.edu.cn/research/soilw#download), and the GOLUM-CNP (v1.0) (Wang et al. 2018(ref50)).
The global maps of modelled N deposition are obtained CICERO-OsloCTM2 (https://igacproject.org/activities/atmospheric-chemistry-climate-model-

intercomparison-project-accmip), GISS-E2-1-G (https://igacproject.org/activities/atmospheric-chemistry-climate-model-intercomparison-project-accmip), THQ (https://esg.pik-potsdam.de/projects/isimip/), and EMEP (https://thredds.met.no/thredds/catalog/data/EMEP/Articles_data/Schwede_etal_Ndep_2018/catalog.html).
The global forest cover map is obtained from http://www.earthenv.org/landcover.

Field-specific reporting

Please select the one below that is the best fit for your research. If you are not sure, read the appropriate sections before making your selection.

☐ Life sciences ☐ Behavioural & social sciences ☒ Ecological, evolutionary & environmental sciences

For a reference copy of the document with all sections, see nature.com/documents/nr-reporting-summary-flat.pdf

Ecological, evolutionary & environmental sciences study design

All studies must disclose on these points even when the disclosure is negative.

Study description	We analyzed results from nine ecosystem-scale paired 15N-labelling experiments in temperate, sub-tropical, and tropical forests across China, and four from temperate Europe and North America, to determine forest N retention fractions for deposited ammonium and nitrate and carbon sink induced by nitrogen deposition.
Research sample	Plant (leaf, branch, stem, and root) and soil (organic soil layer and mineral soil) were sampled. These samples represent the major ecosystem pools of the studied forests where deposited N is retained.
Sampling strategy	The major ecosystem pools including plant component were sampled from within experimental plots consisting of three to five replicate (except at the Harvard forests where single large plots were used). Soil samples were taken from multiple points within the plots. Sample sizes were determined based on availability of resources (costs of 15N tracer and laboratory analysis) and common practices in the field.
Data collection	Plant and soil samples were over-or air-dried to constant weight before grinding. Powdered samples were analyzed in laboratories for N and 15N contents using elemental analyzer-isotope ratio mass spectrometry. Data were recorded using worksheets in Excel. Data were recoded by Dr. S. Li, Dr. A. Wang, W. Zhou, Dr. W. Liu, and Dr. J. Liu for the Chines sites. Knute J. Nadelhoffer, N. Buchmann, and Z. Feng were responsible for the recoding of data from the two Harvard forests in the USA, the Solling site in Germany, and the Wülfersreuth in Germnay, respectively.
Timing and spatial scale	Data collection was conducted between March 1991 and May 2017. Given the multiple sites included in the study, the specific frequency and periodicity of the sampling varied among the 13 sites. The sampling was done at least twice (before and approximately one year after the 15N labeling to quantify one-year retention of th added 15N tracers).The sampling plot also varied among the sites, ranging from 40-450 square meter in size (≥ 100 square meter at 12 of the 13 sites).
Data exclusions	No data we excluded during the analsysis.
Reproducibility	The 15N labelling experimental plots were replicated all sites except at the two Harvard forests to draw conclusions based on a representative samples. At the Harvard forests, for each treatment, 18 small subplots (5 m × 5 m) were used to sample representative samples of the big plots (30 m × 15 m).
Randomization	For each treatment, samples were collected from experimental plots that were randomly allocated to each treatment at the before the 15N labeling. Furthermore, sampling points within each plot were randomly selected for soil sampling. Individual plants of each dominant trees and under-story plants were also selected randomly.
Blinding	Blinding was not relevant for the study because data collection requires a subjective sampling design in the field.

Did the study involve field work? ☒ Yes ☐ No

Field work, collection and transport

Field conditions	There was no specific study condition for the field work.
Location	The study sites are located in China (nine sites), Europe (two), and the USA (two). The locations (Latitude/Longiture) of the field work 127° 34'E, 45° 20'N/117° 46'E, 27° 51'N/101° 15'E, 21° 56'N/108° 51'E, 18° 45'N/108° 53'E, 18° 44'N/106° 41'E, 29° 37'N/124° 54'E, 41° 51'N/124° 55'E, 41° 51'N/128° 28'E, 42° 24'N, 12° E, 50° N/9° 34' E, and 51° 31'N/72° 10'W.
Access & import/export	No written permission was required to conduct the field work as established experimental plots were used at each study site.
Disturbance	No disturbance was caused as designated experimental plots were used.

Reporting for specific materials, systems and methods

We require information from authors about some types of materials, experimental systems and methods used in many studies. Here, indicate whether each material, system or method listed is relevant to your study. If you are not sure if a list item applies to your research, read the appropriate section before selecting a response.

Materials & experimental systems

n/a	Involved in the study
☒	☐ Antibodies
☒	☐ Eukaryotic cell lines
☒	☐ Palaeontology and archaeology
☒	☐ Animals and other organisms
☒	☐ Human research participants
☒	☐ Clinical data
☒	☐ Dual use research of concern

Methods

n/a	Involved in the study
☒	☐ ChIP-seq
☒	☐ Flow cytometry
☒	☐ MRI-based neuroimaging
